# Miniscrew-assisted rapid palatal expansion (MARPE): Factors influencing planning

**DOI:** 10.1590/2177-6709.29.3.e242439.oar

**Published:** 2024-08-09

**Authors:** Cristiane Barros ANDRÉ, Bruno de Paula Machado PASQUA, Gustavo de Andrade JACQUIER, Fabio Dupart NASCIMENTO

**Affiliations:** 1 University of Mogi das Cruzes, Technology Research Center (Mogi das Cruzes/SP, Brazil).; 2 University of São Paulo, School of Orthodontics (São Paulo/SP, Brazil).; 3 Aparecida de Goiânia City College, Department of Radiology (Goiânia/GO, Brazil).; 4 Federal University of São Paulo, Department of Biochemistry, Molecular Biology Division (São Paulo/SP, Brazil).

**Keywords:** Cone-beam computed tomography, Orthodontic anchorage procedures, Orthodontic appliances, fixed, Orthodontic appliance design, Tomografia computorizada de feixe cônico, Procedimentos de ancoragem ortodôntica, Aparelhos ortodônticos, Fixo, Design de aparelhos ortodônticos

## Abstract

**Objective:**

This cross-sectional study evaluated the bone thickness on mini-implants insertion site, the factors that influence the digital planning of MARPE appliance (miniscrew-assisted rapid palatal expansion), and its different designs.

**Methods:**

A total of 135 plannings were assessed regarding the size of the expander screw used, the positioning and the type of the mini-implant rings, and their location in relation to the teeth. Bone thickness measurements were assessed in the region of the mini-implants’ trajectory. Differences between the sexes was verified using the ANOVA test (5% significance).

**Results:**

73 cases were planned with 4 mini-implants and 62 cases, with 6 mini-implants. In 90% of cases, teeth #16 and #26 were used as supports, and the most used expander screw was 13mm (64.1% of cases). The anterior mini-implants of conventional MARPE showed more pronounced insertion in bone in males (5.9 ± 2mm; *p*= 0.025). The extra mini-implants (anterior region) were inserted with greater bone thickness in males (11.1 ± 2.3mm) compared to females (9.9 ± 1.8mm; *p*=0.041). A greater bone thickness was observed in males (10.1 ± 2.1 mm) when using mini-implants in the paramedian region.

**Conclusion:**

Additional rings allow more pronounced bone insertion. Male patients had greater bone thickness, which may be related to greater difficulty in opening the sutures. The alveolar process region seems to be a satisfactory site for mini-implants to those patients with reduced bone thickness in the paramedian posterior region. MARPE appliance must be customized for each patient, due to bone thickness and anatomical variations.

## RESUMO

**Objetivo:**

Avaliar os fatores que influenciam o planejamento digital do expansor MARPE (*miniscrew-assisted rapid palatal expansion*), a espessura óssea no local de inserção dos mini-implantes, e os diferentes possíveis desenhos desse aparelho.

**Métodos:**

Foram avaliados 135 planejamentos quanto ao tamanho do parafuso expansor utilizado, posicionamento, tipo dos anéis dos mini-implantes, e sua localização em relação aos dentes de apoio. As medidas de espessura óssea foram avaliadas na região de inserção dos mini-implantes. As diferenças entre os sexos foram verificadas por meio do teste ANOVA (5% de significância).

**Resultados:**

Os planejamentos tiveram uma distribuição de 73 casos com quatro mini-implantes e 62 casos com seis mini-implantes. Em 90% dos casos, os dentes #16 e #26 foram usados como dentes de apoio, e o parafuso expansor mais usado foi o de 13 mm (64,1% dos casos). Os mini-implantes anteriores do MARPE convencional mostraram uma inserção com maior espessura óssea em homens (5,9 ± 2mm; *p*= 0,025). Os mini-implantes extras (região anterior) foram inseridos com maior espessura óssea nos homens (11,1 ± 2,3 mm), em comparação com as mulheres (9,9 ± 1,8 mm; *p*=0,041). Foi observada maior espessura óssea em homens (10,1 ± 2,1 mm) na região paramediana.

**Conclusões:**

Anéis adicionais permitem uma inserção óssea mais pronunciada. Os pacientes do sexo masculino apresentaram maior espessura óssea, o que pode estar relacionado à maior dificuldade de abertura das suturas.

## INTRODUCTION

Miniscrew-assisted rapid palatal expansion (MARPE) is a technique performed in adults with transverse maxillary deficiency in an outpatient setting. This appliance is industrialized, and variations in design occur only by changing its anteroposterior positioning.^1^

However, for patients with anatomical variations, such as a deviated septum, bone failures, alveolar extension of the maxillary sinus, enlarged nasopalatine duct, sinuous sutures, and even a lack of space in the maxilla, customized appliance design and three-dimensional planning are necessary.^2^ ^,^ ^3^ This digital planning includes the virtual installation of the MARPE and mini-implants, which is performed using tomographic images superimposed on the intraoral scan.^3^ Moreover, this technique provides the means to use segmented appliances, that is, it allows assembling them in individual parts, respecting possible anatomical variations in each patient. Therefore, the expander screw and mini-implant rings (with holes for inserting the mini-implants) are installed virtually, and are responsible for reproducing the digital planning in the oral cavity, and guiding the placement of mini-implants. This versatility allows the addition of more mini-implants to conventional MARPE, such as in cases of reduced bone thickness.^4^

A detailed and in-depth anatomical analysis revealed the need for new resources, such as the use of mini-implants in the alveolar process region.^5^ Although the paramedian region generally has sufficient available bone,^6^ the posterior maxilla often has reduced bone thickness, which prevents adequate bone support. Hence, the possibility of using MARPE has expanded even in patients with mutilations or reduced bone volume in the parasutural region.

Virtual placement and new customization options for MARPE have recently emerged, aiming to increase case predictability and safety. As these are recent tools, the present cross-sectional study used tomography and intraoral scanning to evaluate the factors that influence MARPE planning and different possible designs of this appliance.

## MATERIAL AND METHODS

### STUDY DESIGN

This cross-sectional study analyzed the variations in MARPE designs by the assessment of 135 treatment plans of patients with a median age of 27.3 years (10.8-59.5) of both sexes.

### ETHICAL APPROVAL

This study was approved by the Ethics Committee of the University of Mogi da Cruzes (CAAE: 55356821.1.0000.5497; approval number: 5.360.812). Informed consent was not required because all cone-beam computed tomography (CBCT) images were obtained in the past and used for MARPE planning - which are part of an existing database (Kika Digital Orthodontics Company, Brazil). All data were anonymized before the investigation.

### INCLUSION AND EXCLUSION CRITERIA

The inclusion criteria were: patients of both sexes, with a transverse maxillary deficiency, aged between 10 and 59 years, with CBCT scans performed between October 2022 and June 2023. The exclusion criteria were: CBCT scans of patients with nasopalatine clefts, syndromes, and craniofacial malformations.

### MEASUREMENTS

The CBCT scans were previously obtained using an iCAT equipment (Imaging Sciences International, Hatfield, Pennsylvania) with the following settings: 120 kVp, 18 mA; exposure time = 8.9 s; voxel size = 0.2 mm; and a field of view = 160 × 60 mm.

### VARIABLES EVALUATED

**Figure 1:**
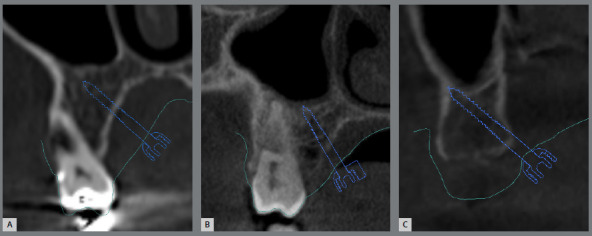
A) Monocortical, B) bicortical, and C) tricortical positioning.

**Figure 2:**
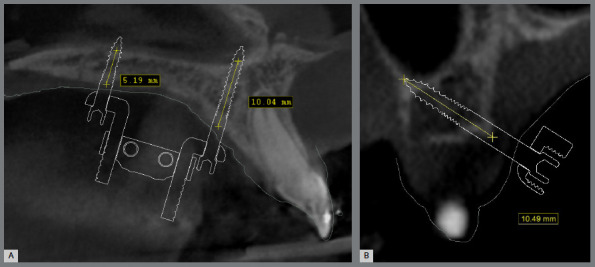
Bone thickness measurement at mini-implant trajectory: **A**) paramedian region (sagittal section), **B**) alveolar bone region (parasagittal section).

A total of 135 plans were evaluated to collect data on the prevalence of expander screw sizes used, the positioning and type of mini-implant rings, bone thickness at mini-implant trajectory, their location in relation to the teeth and transverse palatine suture, the positioning of the mini-implants in relation to the palatal rugae, and their cortical positioning (Fig. 1). The specific points for measuring bone thickness involved identifying the limits of mini-implant insertion along its trajectory, precisely at the midpoint of the implant (Fig. 2). All measurements were conducted by a single operator, calibrated to ensure impartiality and accuracy.

**Figure 3:**
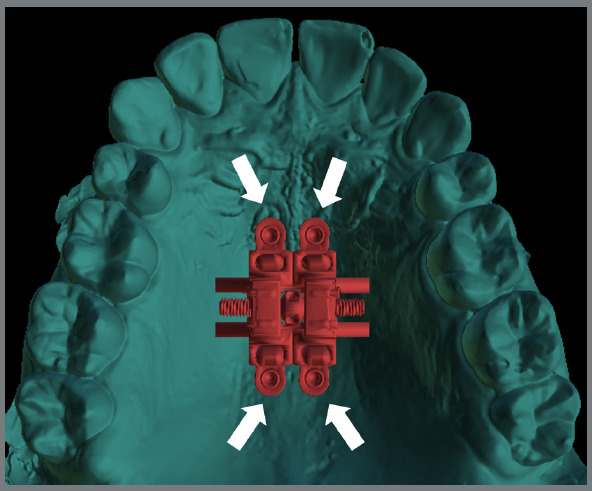
Conventional miniscrew-assisted rapid palatal expansion (MARPE). Note the mini-implants inserted into the rings of this prefabricated appliance.

Industrialized MARPE appliances have four rings that guide the mini-implant insertion. These rings are round and smooth inside, and a specific mini-implant for the MARPE technique is inserted after the appliance has been cemented (Fig. 3). These rings do not keep the mini-implant locked to the appliance in sagittal mechanics, as in cases of MARPE associated with reverse traction of the maxilla or distalization of the posterior teeth. This is because if the banded teeth are moved anteroposteriorly, the appliance can move until it compresses the palatal mucosa.

**Figure 4:**
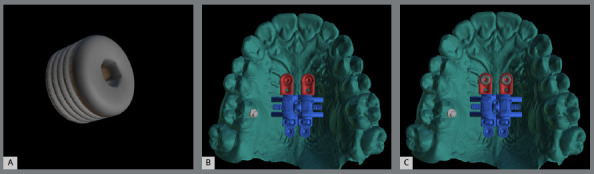
A) Lid of the skeletal ring, which locks the mini-implant to the device. Skeletal ring (red) with mini-implants inserted in the anterior region without the lid (B), and with the lid (C).

**Figure 5:**
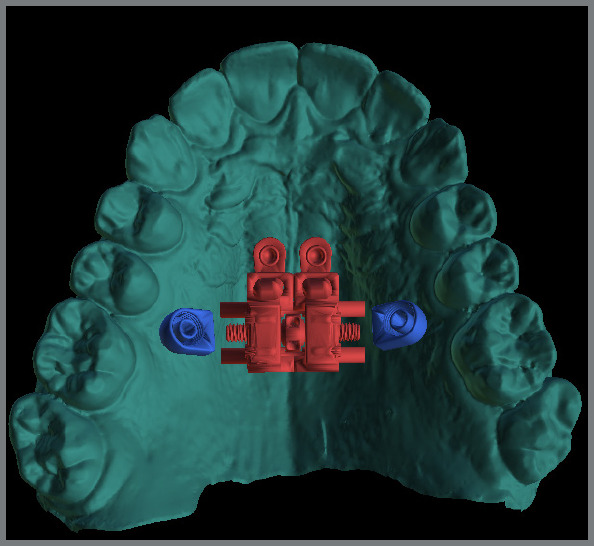
Skeletal rings in the alveolar region.

To solve this problem and broaden the range of biomechanical options, the skeletal ring was developed (PecLab, Belo Horizonte, Brazil), which differs from the conventional ring (hole) due to the internal thread that, after cementing the appliance and placing the mini-implant, receives a lid also with a thread, which guarantees the stability of the appliance for any movement made (Fig 4). The skeletal ring is also planned in the alveolar process in cases where there is reduced bone thickness in the posterior paramedian region (Fig 5). 

**Figure 6:**
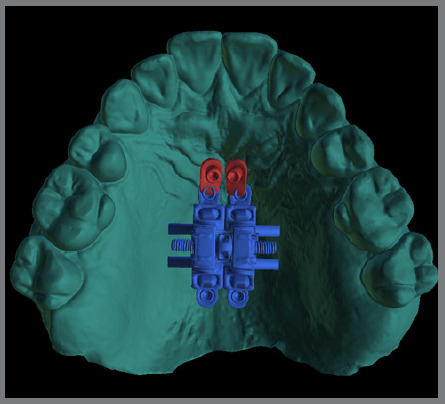
Anterior extra rings.

**Figure 7:**
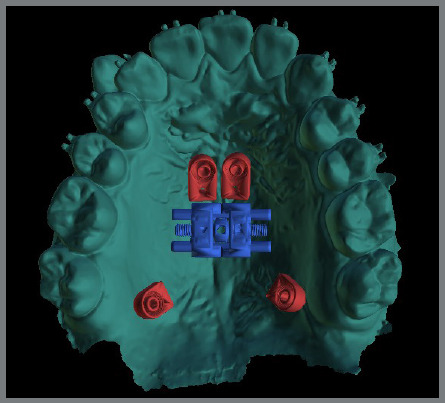
Skeletal rings in the paramedian and alveolar process regions.

Considering other possible MARPE designs, the paramedian region presents four planning options:

1) Four mini-implants from the appliance itself (conventional MARPE, Fig. 3).
2) Four or six mini-implants, two or four from the appliance itself, and two conventional extras (without internal threads) (Fig. 6).
3) Two extra mini-implants in the skeletal rings (with an internal thread) (Fig. 4).
4) Two extra mini-implants in skeletal rings in the anterior region, and two in the posterior region (alveolar process bone) (Fig. 7).

### CBCT MEASUREMENTS

The bone thickness was measured in the region of the mini-implant’s trajectory, in the sagittal sections of each mini-implant. Before the measurements, the CBCT files were renamed with a coded identification number, in the BlueSky software (Blueskybio.com, Blue Sky Bio^®^), to avoid bias. The anatomical features of the maxillary region were assessed through CBCT scans using the BlueSky software (Blueskybio.com, Blue Sky Bio^®^), a reference software for guided surgery protocols.^7^ The orientation of the head position was determined based on a previous methodology.^8^

### STATISTICAL ANALYSIS

Regarding the reproducibility and repeatability of the measurements obtained from the CBCT scan measurements (bone thickness at the mini-implant installation site), they were repeated in 20% of the total sample four weeks after the first measurement. The method error was then calculated using the concordance correlation coefficient.^9^ A significance level of 5% was adopted.

The Shapiro-Wilk normality test was applied to all data. The variables showed a normal distribution and homogeneity (data and histograms were considered); therefore, the mean and standard deviation were used to describe the data.

The sex distribution of the patients was described using absolute and relative frequencies, and the difference between the sexes was verified using the chi-squared tests. 

Nominal variables were described using absolute and relative frequencies, and the difference in their distributions between sexes was verified using Mann-Whitney tests. For the numerical variables, the difference between the sexes was verified using analysis of variance with Bonferroni correction.^9^ For the bone thickness measurements taken from the same patient on both sides, the average between the right and left sides was considered.

Analyses were conducted using IBM-SPSS for Windows software version 20.0, tabulated using Microsoft-Excel software version 2309, and the tests were performed at a 5% significance level.

## RESULTS

### METHOD ERROR

The average correlation coefficient obtained for the tomographic measurement variables evaluated in this study was 0.918 ± 0.17.

### INITIAL DATA, PREVALENCE AND SEX COMPARISON

Only age showed a non-normal distribution (Table 1). No statistically significant differences were observed in the distribution of the patients evaluated, in terms of sex and age. The median age of the 135 patients was 27.3 years (10.8-59.5), comprising 74 men and 61 women with a median age of 27.1 (12.1-59.5) and 24.3 (10.8-55.3) years, respectively (Table 1).

**Table 1:** Distribution of sex, age, and general aspects of MARPE.

	Both n (%)	Female n (%)	Male n (%)	P
Sex: n (%)	135 (100)	61 (45.2)	74 (54.8)	0.263
Age - median (min-max)	27.3 (10.8 - 59.5)	24.3 (10.8 - 55.3)	27.1 (12.1 - 59.5)	0.277ꭞ
GENERAL ASPECTS OF MARPE				
Support teeth for MARPE				0.086
First permanent molars	122 (90)	52 (42.6)	70 (57.4)	
Second permanent molars	6 (4.4)	6 (100)	0	
First right permanent molar, second left permanent molar	3 (2.2)	1 (33.3)	2 (66.7)	
First left molar, second right molar	3(2.2)	2 (66.7)	1 (33.3)	
Second premolars	1 (0.7)	0	1 (100)	
Expander screw size				0.184
11mm	48 (35.6)	18 (37.5)	30 (62.5)	
13mm	87 (64.1)	43 (49.4)	44 (50.6)	
Quantity of mini-implants				0.733
4	73 (54.1)	32 (43.8)	41(56.2)	
6	62 (45.9)	29 (46.8)	33 (53.2)	
MARPE WITH SKELETAL RINGS				
Reason for using skeletal ring				0.606
Ensure anterior positioning from the transverse palatine suture	45 (33.3)	23 (51.1)	22 (48.9)	
Reduced bone thickness in the posterior region	43 (31.9)	17 (39.5)	26 (60.5)	
Generalized reduced bone thickness	6 (4.4)	2 (33.3)	4 (66.7)	
Class III patient	39 (28.9)	18 (46.2)	21 (53.8)	
Thick mucosa	1 (0.7)	1 (100)	0	
Agenesia	1 (0.7)	0	1 (100)	
Skeletal rings location				0.783
Alveolar process	56 (41.5)	23 (41.1)	33 (58.9)	
Paramedian region	51 (37.8)	27 (52.9)	24 (47.1)	
Both	28 (20.7)	11 (39.3)	17 (60.7)	
Exact position of the skeletal rings				0.985
Between premolars	0	0	0	
Between second premolars and first permanent molars	36 (41.9)	20 (55.6)	16 (44.4)	
Between molars	31 (36)	9 (29)	22 (71)	
Distal from second molars	19 (22.1)	7 (36.8)	12 (63.2)	

ꭞ Mann-Whitney test.

*p<0.05; **p<0.01; ***p<0.001; ****p<0.0001.

### GENERAL ASPECTS OF MARPE

No statistically significant differences between the sexes were observed in terms of the teeth used to support the device. In 90% of cases, maxillary first molars were used as supports, and second molars were used, with teeth #17 and #26 or teeth #26 and #17, to a lesser extent. Them second premolars were used in only one case.

The most commonly used expander screw was 13 mm in diameter (64.1% of cases), with no significant differences between the sexes.

Planning with 4 and 6 mini-implants had a homogeneous distribution, with 73 and 62 cases with 4 and 6 mini-implants, respectively, without significant differences between the sexes.

The reasons for choosing skeletal rings varied greatly, with 33.3% (Table 1) ensuring that the mini-implants were positioned anterior to the transverse palatine suture. Due to the reduced bone thickness in the posterior region (31.9%) and Class III cases (28.9%) (Table 1), these patients received skeletal rings, with no significant differences between the sexes.

The skeletal rings were most frequently located in the alveolar process (41.5%), followed by the paramedian region (37.8%). In 28 patients in whom a skeletal ring was planned (20.7%), the location was both paramedian and alveolar (Table 1). 

Many skeletal rings were planned between the second premolars and first molars (41.9%). In 36% of cases, the skeletal rings were positioned between the molars, and in a minority of cases (22.1%), they were positioned distal to the second molars.

### BONE THICKNESS AND CORTICAL POSITIONING AT THE MINI-IMPLANT SITE

A greater thickness of bone was observed when comparing men (10.1 ± 2.1 mm) with women (9.4 ± 2.1 mm) (Tab. 2), for the cases of mini-implants with skeletal rings in the paramedian region; without statistically significant difference. Although the prevalence of bicorticality was higher in men (*p*=0.003) (Table 2). 

**Table 2:** Distribution of positioning in relation to the cortical bone. Bone thickness within the mini-implant insertion area (comparison between the sex - ANOVA test with Bonferroni correction).

	Both	Female	Male	SE	P
MINI-IMPLANTS ASSOCIATED WITH THE SKELETAL RING					
Bone thickness - Paramedian region (mm)	9.8 ± 2.1	9.4 ± 2.1	10.1 ± 2.1	0.2	0.091
Positioning of the mini-implants - Paramedian region: n(%)					0.003**
Monocortical	28 (18.2)	15 (53.6)	13 (46.4)		
Bicortical	126 (81.8)	57 (45.2)	69 (54.8)		
Bone thickness - Alveolar Process region (mm)	8.6 ± 2.9	8.1 ± 2.7	8.9 ± 2.9	0.2	0.093
Positioning of the mini-implants - Alveolar Process: n(%)					0.628
Monocortical	34 (20)	9 (26.4)	25 (73.6)		
Bicortical	116 (68.2)	47 (40.5)	69 (59.5)		
Tricortical	20 (11.8)	14 (70)	6 (30)		
Bone thickness extra skeletal ring - Anterior region (mm)	9.3 ± 0.7	8.7 ± 0.3	9.5 ± 0.7	0.2	0.211
Positioning of extra skeletal ring - Anterior region: n(%)					0.564
Monocortical	3 (37.5)	1 (33.3)	2 (66.7)		
Bicortical	5 (62.5)	1 (20)	4 (80)		
MIN-IMPLANTS WITHOUT SKELETAL RINGS					
Anterior mini-implant bone thickness (mm)	5.6 ± 2.1	5.1 ± 2.2	5.9 ± 2	0.2	0.025*
Positioning the anterior mini-implant: n(%)					0.412
Monocortical	3 (2.8)	2 (66.7)	1 (33.3)		
Bicortical	103 (97.1)	44 (42.7)	59 (57.3)		
Posterior mini-implant bone thickness (mm)	3.31 ± 1.2	3.3 ± 1.1	3.3 ± 1.4	0.1	0.912
Positioning the posterior mini-implant: n(%)					1.000
Monocortical	0	0	0		
Bicortical	172 (100)	88 (51.2)	84 (48.8)		
Extra mini-implant bone thickness - anterior region (mm)	10.6 ± 2.2	9.9 ± 1.8	11.1 ± 2.3	0.3	0.041*
Positioning the extra mini-implant - Anterior region: n(%)					0.001**
Monocortical	26 (46.4)	4 (15.3)	22 (84.6)		
Bicortical	30 (53.6)	18 (60)	12 (40)		

*p<0.05; ** *p* <0.01; ***p<0.001; ****p<0.0001.

Mini-implants with a skeletal ring in the alveolar region presented bone thickness of 8.6 ± 2.9 mm in both sexes. The frequencies of positioning were 20% monocortical, 68.2% bicortical, and 11.8% tricortical, with no differences between the sexes (Table 2). 

The anterior mini-implants (conventional MARPE) showed greater insertion in bone in men (5.9 ± 2mm), with a statistically significant difference (*p*= 0.025). There was no difference between the sexes in terms of cortical positioning, with most cases being bicortical (97.1%) (Table 2).

All posterior mini-implants were bicortical, and the sex distribution was homogeneous. The thickness of the bone in the mini-implant region was also homogeneous (3.31 ± 1.2 mm) (Table 2).

The extra mini-implants (anterior region) were inserted with greater bone thickness in men (11.1 ± 2.3 mm) compared to women (9.9 ± 1.8 mm), with a statistically significant difference (*p*=0.041) (Tab. 2). Female patients had a significantly higher frequency of bicortical positioning than that in men (*p*=0.001).

## DISCUSSION

Understanding the possibilities of appliance design for the MARPE technique is important because we can observe numerous anatomical variations during digital planning.^3^ Therefore, this study aimed to present and discuss the factors that influenced the design of these appliances. 

The size of the expander screw used can vary depending on the design possibilities. From the data obtained in the present study, patients were more likely to use a 13-mm expander screw (64.1%; Table 1), suggesting that most patients required a large amount of maxillary expansion.

About the support teeth, according to the existing literature,^1^ ^,^ ^10^ the bands are made in the first upper molars, as was the case in most patients in this sample (90%) (Table 1). However, 10% of the patients presented mutilation, indicating that it was necessary to alter the appliance’s supporting teeth and modify its design.

The conventional MARPE appliance has four parasutural mini-implants, and is positioned vertically and centered on the maxilla (Fig 3). According to Lee et al.,^10^ teeth should be used as a reference for positioning the appliance, that is, the MARPE should be positioned in the first premolars and first molars. This design does not consider the most anterior region of the maxilla, which has pronounced bone thickness;^11^ ^,^ ^12^ therefore, skeletal support may be inadequate (minor bone thickness). In the present study, the region of the anterior mini-implants had greater bone thickness in men (5.9 ± 2 mm, Table 2), with a statistically significant difference (*p*=0.025). The mini-implants were positioned bicortically in both sexes, which is critical for the MARPE technique’s success.^13^ ^,^ ^14^ An important feature of conventional MARPE is that the positioning of the anterior mini-implants must be performed with caution, as its design has a fixed distance between the anterior and posterior mini-implants, which can lead to a collision with the nasopalatine duct or the transverse palatine suture.^3^

Bone thickness in the posterior paramedian region had a mean thickness of 3.31 ± 1.2 mm, which required additional rings to increase skeletal anchorage (six mini-implants), as the MARPE with four mini-implants would not provide adequate anchorage. In other words, the bone thickness in the posterior paramedian region influences the use of four or six mini-implants. In 100% of the cases, additional rings were placed with bicortical positioning in male and female patients. However, this does not imply the success of the technique because the thickness could be reduced in this region, as discussed earlier. This occurred in 31.9% of patients (Table 2), which calls for attention, as there may be side effects on the supporting teeth, bone loss around the mini-implants, and failure due to this loss.^15^

The design with additional mini-implants in the anterior maxillary region (Fig 6) is indicated to increase anchorage and chances of success of cases, by ensuring two more skeletal anchorage areas in the anterior region, in a region with adequate bone volume in the anterior nasal spine area.^4^ In this region, there is a greater bone thickness in male patients (approximately 0.4 mm) (Table 2). Consequently, female patients showed a statistically significant difference in bicortical positioning in this region, due to the reduced bone volume. In 49.5% of the evaluated cases, the appliances were modified with additional mini-implants due to reduced or increased bone thickness (torus palatinus).^3^

When comparing the bone thickness of the mini-implants between the conventional MARPE (5.6 ± 2.1 mm) and using additional rings (10.6 ± 2.2 mm, Table 2), the anterior paramedian region towards the anterior nasal spine had a greater bone volume. Thus, planning with four and six mini-implants had a homogeneous distribution, which might indicate that 50% of the patients had altered bone thickness, and MARPE with six mini-implants was indicated.

A design with skeletal rings in the anterior region (37.8% of cases; Table 2) (Fig 4) was used in cases with reduced anteroposterior size of the maxilla, ensuring that the mini-implants were positioned anterior to the transverse palatine suture (33%) (Table 1). In cases of increased volume of the nasopalatine foramen, mini-implants can be inserted angled, to avoid colliding with the structure. At this site, male patients had greater bone thickness, as observed with additional mini-implants. There was also a higher frequency of bicortical positioning in male patients. The great advantage of skeletal rings is in combined mechanics, such as reverse traction of the maxilla and expansion, which benefits from the use of the skeletal ring, due to the locking of the mini-implants to the appliance, preventing any dental side effects of mesialization, movement from the expander screw to the anterior, and inflammation of the mucosa in this region.

Skeletal rings can also be used in the alveolar process (Fig. 5), where these mini-implants were inserted in 41.5% of cases, with a bone thickness of 8.6 ± 2.9 mm, 20% of which were monocortical, 68.2% bicortical and 11.8% tricortical. The site of placement of these mini-implants should be selected carefully, assessing the bone volume, mucosal thickness, space between tooth roots, mini-implant insertion axis, and location of the greater palatine artery.

In the designs with skeletal rings in the alveolar process, the majority were positioned between the second premolars and first molars (41.9%), followed by the position between molars (36%). In a minority of cases (22.1%), the skeletal rings were positioned distal to the second molars, due to the lack of an insertion axis of the device or bone volume, with only 11.8% of mini-implants being tricortical, 20% of cases were monocortical, and the highest frequency was bicortical positioning (68.2%). The bicortical positioning of mini-implants allows for a broader distribution of expansion forces, preventing the concentration of stress areas around the mini-implants, and resulting in improved skeletal changes.^13^ ^,^ ^14^ This variation in prevalence between bicortical and monocortical positioning is attributed to factors such as root placement, bone volume, and the range of mini-implant sizes available in the market.^12^

In complex cases, the anterior and alveolar process skeletal rings can be combined (Fig 7). The combination of skeletal rings in the alveolar process and additional anterior rings occurred in 20.7% of the cases planned with a skeletal ring. This suggests a high frequency of more complex cases in this study, where reduced posterior bone thickness, with a reduced anteroposterior limit was noted, not allowing the mini-implants to be placed before the transverse palatine suture, with the need for angulation of the posterior mini-implants or even with reduced stability for maxillary protraction.

Although the conventional MARPE appliance is more likely to be bicortical, it is not installed in a region with good bone volume, due to the anteroposterior limit. Specifically, the mini-implants can touch the nasopalatine nerve and duct, as well as the transverse palatine suture, if not planned three-dimensionally. Additional mini-implants are an excellent tool for cases of varying bone volume and an enlarged nasopalatine duct; however, in cases of maxillary protraction, associated distalization, or reduced parasutural bone thickness, it is ideal to use skeletal rings (skeletal ring stabilizes the appliance, preventing collision with soft tissue or loss of anchorage). 

In this study, we illustrated that the design of the appliance can vary according to the amount of expansion, supporting teeth, the thickness of soft and hard tissues, anatomical variations such as palatal torus, and increased diameter of the nasopalatine duct. Thus, the MARPE appliance must be customized for each patient, and guided digital planning must be performed for safe and predictable treatment. 

## CONCLUSIONS

» Prefabricated appliances have limitations in providing the amount of bone thickness in the anterior region (anteroposterior distance already defined), compared to the appliance with additional rings.
» Additional rings, whether with or without internal threads, make it possible to be inserted in more bone, due to the possibility of customization. Additionally, they prevent collisions with important structures.
» Mini-implants int the alveolar process region seems to be a satisfactory option to patients with reduced bone thickness in the paramedian posterior region.
» Male patients had greater bone thickness than female, which may be related to greater difficulty in opening the sutures. MARPE appliances must be customized for each patient, due to several potential anatomical variations. Guided digital planning should be conducted to ensure safety and predictability.
